# Management of Pressure Sore at Tertiary Care Center in Western Nepal: An Observational Study

**DOI:** 10.31729/jnma.v64i293.9303

**Published:** 2026-01-31

**Authors:** Piyush Giri

**Affiliations:** 1 Department of Surgery, Pokhara Academy of Health Sciences, Pokhara, Kaski, Gandaki, Nepal

**Keywords:** *chronic wounds*, *pressure sore*, *pressure ulcer*

## Abstract

**Introduction:**

Pressure sore are localized skin and soft tissue damage typically occurring over bony prominences due to impaired blood supply from sustained pressure. This study aims to review the clinical profile of patients with pressure sore and the type of management of pressure sore in regional referral center of western Nepal.

**Methods:**

This retrospective, observational study was conducted at the Department of Burns, Plastic and Reconstructive Surgery, Charak Memorial Hospital, Pokhara, from January 2023 to December 2024. Ethical approval was obtained from National Health Research Council on 16 February 2025 (Reference no:1839). The study included all patients regardless of age and gender who were treated for pressure sores during the study period, with complete medical records. Patient demographics, sore characteristics, predisposing factors, and treatment modalities were analyzed.

**Results:**

There were 21 patients with 42 pressure sore wounds, included with mean age 45.14±25.54 years (range: 16-92 years). Of all patients 16 (79.19%) were male and 16 patients (76.19%) were patients from outside Pokhara Valley. Spinal cord injury was present in 12 (57.14%) patients. The sacral and trochanteric regions were affected with 14 (33.33%) wounds each. There were 26 (61.90%) wounds classified as National Pressure Ulcer Advisory Panel Stage IV. Surgical management was performed in 14 (66.67%) patients with Local flap was used to reconstruct 12 (50%) of the wounds.

**Conclusions:**

Pressure sore in this population predominantly affected middle-aged males with spinal cord injuries, presenting with advanced-stage wounds. Local pattern flap was the most common method of soft tissue coverage.

## INTRODUCTION

Pressure sore is localized damage to the skin and underlying soft tissue, usually over the bony prominences, caused by tissue placed under pressure sufficient to impair the blood supply to the area. Risks are higher in bedridden or wheelchair-bound patients.^1,2^

The National Pressure Ulcer Advisory Panel (NPUAP) 2016 stages the wound according to depth of tissue involved.^3^ Pillars for managing pressure ulcers are nutrition, repositioning of patients, and mobilization as well as debridement and wound coverage with flap and skin grafts.^4,5^ For developing countries such as Nepal, little has been studied on the epidemiology and management of pressure ulcers. This study will give us baseline data on the management of pressure ulcers and enable us to give data-driven management in the future.

This study aims to review the clinical profile of patients with pressure sore, characteristics of wound and the type of treatment provided.

## METHODS

This is a retrospective cross-sectional study conducted at the Department of Burns, Plastic and Reconstructive Surgery, Charak Memorial Hospital, Pokhara, Gandaki Province, Nepal. The study reviewed medical records of patients treated for pressure sores over a two-year period from January 1, 2023, to December 31, 2024. Ethical clearance for the study was obtained from the Nepal Health Research Council (NHRC) on February 16, 2025 (Reference number: 1839).

The study included all patients who met inclusion criteria during the study period. Inclusion criteria were patients of any age or gender who were treated for pressure sores during the study period with complete medical records. Patients with missing or incomplete records, patients treated in outpatient clinic, as well as those who refused treatment modality, as well as missing key clinical data record were excluded. Data were extracted from medical records using a structured proforma designed specifically for this study. Demographic variables such as age, sex, residence and comorbidities (including diabetes mellitus, hypertension, and neuropathy) were recorded. Residence was categorized as “inside Pokhara Valley” for patients living within the Pokhara Valley, and as “outside Pokhara Valley” for patients residing in towns and surrounding districts beyond the valley. Patients who had at least one family member consistently attending to them at home were classified as having family support.

Clinical data included pressure sore location (sacrum, ischium, trochanter, heel, etc.), staging based on the National Pressure Ulcer Advisory Panel (NPUAP) classification. Stage 1 wound has intact skin with localized area of non-blanchable erythema. Stage 2 wounds have partial-thickness loss of skin with exposed dermis however fat and deep tissue are not visible. The wound bed is viable, pink or red, moist and may present as blister. Adipose tissue or deeper tissue is not visible. Granulation tissue, slough and eschar, are not present. Stage 3 wound has full thickness loss of skin in which adipose tissue is visible in ulcer and granulation tissue and epibole is present. Deeper tissue such as bone, tendon, ligament, cartilage or bone is not exposed. In stage 4 there is full thickness skin and tissue loss with exposed or directly palpable fascia, muscle, tendon, ligament, cartilage or bone. Slough and/or eschar may be visible. Epibole (rolled edges), undermining, and/or tunneling often occur.

Unstageable full thickness pressure injury wounds are defined as having full thickness skin and tissue loss in which the extend of tissue damage within the ulcer cannot be confirmed because it is obscured by slough or eschar. If slough eschar is removed, a stage 3 or stage 4 pressure injury will be revealed. Deep tissue pressure injury is defined as wounds with full thickness skin and tissue loss in which the extend of tissue damage within the ulcer cannot be confirmed because it is obscured by slough or eschar. Intact or nonintact skin with localized area of persistent non blanchable deep red, maroon, purple discoloration or epidermal separation revealing a dark wound bed or blood filled blisters. Pain and temperature change often precede skin color changes. ^3^

Management details included type of treatment (conservative or surgical), nature of surgical procedures (debridement, flap coverage, skin grafting).

All data were entered and analyzed using Microsoft Excel Version 16.45. Descriptive statistical analysis was performed, and the outcomes were presented in a tabular format. Categorical data were summarized using frequencies and percentages, while continuous variables were described using means and standard deviations.

## RESULTS

The study included 21 patients with a total of 42 pressure sore wounds, with a mean age of 45.14±11 years (range: 16-92 years) There were 16 (76.19%) male and 16 (76.19%) patients were from outside Pokhara valley (Table 1).

Predisposing factors included spinal cord injury in 12 (57.14%) patients while 14 (66.67%) patients were immobile and 18 (85.71%) patients were receiving family support (Table 2).

There were 14 (33.33%) pressure sore wounds present in sacral region and 14 (33.33%) pressure sore wounds in trochanteric region. Out of 42, 26 (61.9%) of wounds were classified as NPUAP Stage IV. There were 37 (88.10%) wounds contaminated with slough while 12 (28.57%) wounds presented with osteomylelitis (Table 3).

Out of 42 wounds, there were 24 (57.14%) wounds managedconservatively. Surgical management wasperformed on 14(66.67%) of patients (Table 4).

Surgical management involved 18 wounds with a total of 24 procedures. There were 5 (17.24%) cases of complication, including wound dehiscence in 4 (80.00%) cases and marginal flap necrosis in 1 (20.00%). Mean hospital stay of patients was 23.26 days ± 19.82, patients in conservatively managed patient was 8.60 ±13.11 days and patient managed with surgery stayed mean 34.1± 31.95 days.

Of the 24 surgical procedures, local flap reconstruction was done in 16 (16.67%) cases and skin graft was done in 3 (12.50%) cases (Table 5).

Regarding pressure sore development, 13 (61.90%) patients developed sore in a home setting, while 8 (38.09%) developed pressure sore in hospice or hospital environments. Preventive measures were present in 10 (47.62%) cases. A history of pressure sores was reported in 5 (23.81%) patients. Among there, recurrence at the same site occurred in 2 (40.00%) patients and at a different site in 3 (60.00%) patients.

**Table 1:** Demographics characteristics of patients with pressure sore (n=21).

Characteristic	n (%)
Male	16(76.19)
Female	5(23.81)
From outside Pokhara Valley	16(76.19)
From inside Pokhara Valley	5(23.81)

**Table 2:** Predisposing factors and patient support in patients with pressure sore (n=21).

Characteristic	n (%)
Spinal cord injury	12(57.14)
Chronic debilitating illness	9(42.86)
Family support present	18(85.71)
Family support absent	3(14.29)

**Table 3:** Pressure sore characteristics and staging based on the National Pressure Ulcer Advisory Panel (NPUAP) classification (n=42).

Characteristic	n (%)
Location	
Sacral region	14(33.33)
Trochanteric region	14(33.33)
Ischial region	6(14.29)
Other regions	8(19.05)
Stage	
Stage II	2(4.76)
Stage III	10(23.81)
Stage IV	26(61.90)
Unstageable	1(2.38)
Deep tissue pressure injury	3(7.14)
Wound characteristics	
Wounds with slough	37(88.10)
Wounds with eschar and slough	4(9.52)
Wounds with slough and granulation	1(2.38)
Osteomyelitis	12(28.57)

**Table 4:** Management of pressure sore (n=42).

Characteristic	n(%)
Wounds operated	18(42.86)
Wounds managed conservatively	24(57.14)

**Table 5:** Modality of surgery of pressure sore (n=24).

Modality of Surgery	n(%)
Local flap	16(66.67)
Skin graft	3(12.50)
Debridement only	2(8.33)
Primary closure	3(12.50)

## DISCUSSION

The demographic profile observed in this study demonstrates patterns that are consistent with existing epidemiological evidence regarding the occurrence of pressure sores. The marked male predominance (76.19% compared to 23.81% female) supports findings from previous studies that report a higher incidence of pressure sores among males, particularly in populations affected by spinal cord injury.^6-9^ This gender difference is largely attributable to the higher incidence of traumatic injuries among males, including road traffic accidents and occupational trauma, which remain leading causes of spinal cord injury in Nepal and similar low- and middle-income settings. The overrepresentation of younger adults aged 21-40 years further aligns with the demographic characteristics of traumatic spinal cord injury. According to the National Spinal Cord Injury Statistical Center, the average age at injury is approximately 43 years, with nearly 78% of new cases occurring among males, a trend reflected in the present cohort. The relatively young age of affected individuals highlights the long-term clinical, social, and economic burden associated with pressure sores, including prolonged morbidity, repeated hospital admissions, reduced productivity, and increased dependence on caregivers.^7-9^

The geographic distribution of patients indicates that 76.19% originated from areas outside the Pokhara Valley, suggesting that the study institution functions as a tertiary referral center for complex wound management and reconstructive surgery. This pattern may reflect disparities in access to specialized care between urban and rural regions of Nepal. Patients from remote areas are more likely to experience delays in diagnosis, limited availability of preventive interventions, and inadequate follow-up, which may contribute to presentation at advanced stages of disease.^8^ Geographic barriers, financial constraints, and limited transportation options may further exacerbate delays in accessing care, particularly for patients requiring repeated evaluations. The findings emphasize the importance of improved referral pathways, strengthening of peripheral healthcare services, and enhanced awareness of pressure sore prevention at the community level.

Spinal cord injury was identified as the primary predisposing factor in 57.14% of cases, a proportion higher than that typically reported in general hospital populations, where chronic debilitating conditions such as stroke, advanced age, and prolonged medical illness are more common.^9-11^ The development of pressure sores in patients with spinal cord injury is multifactorial. Loss of sensation below the level of injury eliminates pain-mediated protective responses, while motor paralysis restricts voluntary repositioning and pressure relief. Autonomic dysfunction further compromises vasomotor control, impairing tissue perfusion and oxygen delivery. In addition, neurogenic bowel and bladder dysfunction, recurrent infections, metabolic alterations, and nutritional deficiencies adversely affect wound healing capacity.^12,13^ The interaction of these factors explains both the severity of pressure sores and the frequent occurrence of multiple wounds in this subgroup.

An important observation in this study was that 33.33% of patients were categorized as mobile, which challenges the conventional assumption that immobility alone is responsible for pressure sore development. This finding suggests that factors such as micromobility, duration of sustained pressure, seating ergonomics, and pressure redistribution may be more critical determinants than gross mobility status.^14^ In ambulatory or wheelchair-dependent individuals, prolonged sitting, inappropriate wheelchair fit, inadequate pressure-relieving cushions, and improper transfer techniques can result in localized tissue ischemia despite apparent mobility. These findings indicate that activity alone is insufficient protection without appropriate education, assistive devices, and pressure management strategies, particularly in individuals with sensory impairment.

The anatomical distribution of pressure sores revealed equal involvement of the sacral and trochanteric regions, reflecting posture-dependent pressure loading patterns described in biomechanical studies. Sacral sores are commonly associated with prolonged supine positioning, frequently encountered in hospitalized or bed-bound patients, whereas trochanteric sores may result from side-lying positions without adequate pressure redistribution. The presence of ischial sores, although less frequent, is clinically significant due to their location in high-pressure, weight-bearing regions.^2,15^ Ischial sores are particularly challenging to manage, as sitting pressures can exceed 200 mmHg, and effective pressure relief is difficult to achieve in wheelchair-dependent individuals. These anatomical considerations often influence both preventive strategies and reconstructive decision-making.^16^

The predominance of Stage IV pressure sores (61.90%) indicates deficiencies in early detection, preventive strategies, and timely intervention.^16^ Advanced-stage pressure sores are characterized by full-thickness tissue loss, often with exposure of muscle or bone, and are frequently complicated by infection and osteomyelitis, necessitating extensive debridement and reconstructive surgery.^5^ The presence of slough in 88.10% of wounds suggests ongoing tissue necrosis and high bacterial burden, features commonly associated with chronic non-healing wounds.^17^ Delayed presentation may further contribute to wound chronicity and complexity. The observation that patients presented with an average of two wounds, with some having as many as five, supports existing evidence that pressure sore development is a systemic process. Individuals who develop one pressure sore are at increased risk of additional lesions due to shared underlying risk factors.^7^

The finding that 61.90% of pressure sores developed at home has important implications for healthcare delivery. This suggests inadequacies in discharge planning, caregiver education, and continuity of care following hospital discharge. Limited access to pressure-relieving equipment, insufficient caregiver training, and lack of structured follow-up contribute to delayed recognition and progression of pressure sores in the community. Studies have shown that community-acquired pressure sores often present at more advanced stages than those identified in institutional settings, resulting in increased morbidity and treatment complexity.^18,19^ These findings highlight the importance of strengthening transitional care and improving post-discharge support for high-risk patients.

Preventive measures were documented in fewer than half of the patients, indicating a significant opportunity for improvement. Evidence-based prevention strategies include regular use of validated risk assessment tools, implementation of systematic repositioning protocols, appropriate selection of support surfaces, meticulous skin care, and optimization of nutritional status.^4,20^ The observation that 60% of recurrences occurred at different anatomical sites suggests that systemic risk factors play a more significant role than localized issues alone. This underscores the importance of comprehensive prevention programs that address overall patient risk rather than focusing solely on individual wound sites. ^21^

Surgical intervention was required in 66.67% of patients and 42.86% of wounds, reflecting the advanced disease burden encountered at this tertiary care center. The predominance of flap reconstruction is consistent with established recommendations for the management of Stage IV pressure sores, as simple closure techniques are associated with high failure and recurrence rates. Flap reconstruction provides durable, well-vascularized tissue capable of withstanding pressure and facilitating healing. The requirement for surgical intervention in all ischial wounds further illustrates the challenges associated with this anatomical region, where persistent pressure and shear forces limit the effectiveness of conservative measures.^22^ The predominance of flap procedures aligns with literature recommendations for Stage IV sores, where simple closure techniques have high failure rates. Ischial wounds reflects the unique challenges of this anatomical location, including the high-pressure environment with pressures exceeding 200 mmHg during sitting, significant shear forces during transfers and repositioning, and limited conservative options due to difficulty achieving complete pressure relief in wheelchair users.^23^

The postoperative complication rate of 17.24%, with wound dehiscence accounting for the majority of complications, is comparable to rates reported in previous studies. Complications are influenced by multiple factors, including poor nutritional status, comorbidities such as diabetes mellitus, smoking, inadequate debridement, excessive tension at closure sites, and continued pressure exposure during the postoperative period. Similar complication rates have been reported by Keys et al., emphasizing the importance of appropriate patient selection and meticulous postoperative care. Extended hospital stays associated with complications reflect the complex and multidisciplinary nature of pressure sore management, encompassing preoperative optimization, surgical expertise, vigilant postoperative monitoring, and structured rehabilitation. The economic burden associated with advanced pressure sores further highlights the importance of effective preventive strategies.^22^

Conservative management was employed in 33.33% of patients, including a proportion with Stage IV pressure sores. This approach was chosen due to patient comorbidities limiting surgical eligibility, palliative care considerations, or informed patient preference after discussion of treatment options. These findings underscore the importance of individualized treatment planning that considers patient factors in addition to wound characteristics.^23^

The substantial difference in hospital length of stay between surgically (34.7 days) and conservatively managed patients (8.6 days) has significant healthcare economic implications. Extended stays reflect the complexity of care required, including pre-operative optimization through nutritional support, infection control, and medical stabilization; surgical complexity involving multiple procedures and staged reconstructions; post-operative monitoring for flap surveillance and complication management; and rehabilitation needs including physical therapy and pressure relief training. Studies by Brem et al. estimated average costs of $43,180 per Stage IV pressure sore with surgical cases significantly exceeding this amount.^24^

This study has limitations, including its single-center design, retrospective methodology, and relatively small sample size, which may limit generalizability. The lack of long-term follow-up data precludes assessment of recurrence rates and longterm functional outcomes. Despite these limitations, the study provides meaningful insight into the patterns, severity, and management of pressure sores in a tertiary care setting in Nepal and highlights the need for improved prevention, early detection, and continuity of care.

## CONCLUSION

Pressure sore predominantly affected middle-aged males with spinal cord injuries who are bed ridden, presenting with advanced-stage wounds. Local pattern flap was the most common method of soft tissue coverage.

## Data Availability

The data are available from the corresponding author upon reasonable request.
